# Treatment of bacterial biothreat agents with a novel purified bioactive lactoferrin affects both growth and biofilm formation

**DOI:** 10.3389/fcimb.2025.1603689

**Published:** 2025-06-17

**Authors:** Christian Xander, Elsie E. Martinez, Ronald G. Toothman, Christina L. Gardner, Ju Qiu, Jonathan Snedeker, Matthew H. Bender, Christopher Hlubb, Crystal W. Burke, Joel A. Bozue, Kevin D. Mlynek

**Affiliations:** ^1^ Bacteriology Division, United States (U.S.) Army Medical Research Institute of Infectious Diseases (USAMRIID), Frederick, MD, United States; ^2^ Virology Division, United States (U.S.) Army Medical Research Institute of Infectious Diseases (USAMRIID), Frederick, MD, United States; ^3^ Regulated Research Administration Division, United States (U.S.) Army Medical Research Institute of Infectious Diseases (USAMRIID), Frederick, MD, United States; ^4^ Lactea Therapeutics, Frederick, MD, United States

**Keywords:** *Francisella*, *Burkholderia*, biofilm, lactoferrin, tularemia, melioidosis, glanders

## Abstract

Lactoferrin is known to exhibit broad spectrum activity against a multitude of bacteria, fungi, and viruses due to its multi-functional mode of action. Recently, Lactea Therapeutics and its affiliates have developed a novel, patent-pending technology to purify naturally derived bovine lactoferrin (Lactea Lf) for use as a medical countermeasure that was not previously available. To assess the efficacy of Lactea Lf against biothreat pathogens, we performed biofilm inhibition assays and generated dose-response curves against *Burkholderia pseudomallei*, *Burkholderia mallei*, and *Francisella tularensis* for proof-of-principle studies. Here, we show that Lactea Lf can significantly inhibit biofilm and decrease the overall growth in a dose dependent manner for all *Burkholderia* species tested. Of note, Lactea Lf was found to completely inhibit biofilm formation by virulent *B. pseudomallei* without observing complete growth inhibition. The growth of *F. tularensis* was also significantly inhibited when cultured in the presence of Lactea Lf and appeared more sensitive to treatment when compared to *B. pseudomallei*. Based on these results, a pneumonic infection model using the *F. tularensis* LVS strain was performed prophylactically administering Lactea Lf and continuing treatment post challenge. No protection was observed in this model which prompted biodistribution studies using fluorescent tagged Lactea Lf. These experiments revealed that therapeutic material was mainly confined to the NALT region following intranasal delivery and then quickly dispersed or inactivated suggesting that future formulation and delivery method could be addressed to increase *in vivo* treatment efficacy. Taken together, these data support that Lactea Lf is a potentially new candidate for further studies as a broad-spectrum antimicrobial medical countermeasure with efficacy against several high priority biodefense-related bacterial pathogens.

## Introduction

Mammals rely on their innate immune system as a first line of defense to protect against infectious diseases. One component of the innate immune system that helps combat these insults is the glycoprotein lactoferrin. Lactoferrin is an iron-binding glycoprotein that is both expressed in neutrophils as well as in many mammalian secretory fluids, such as saliva, milk, and tears (Cao et al., 2022). Lactoferrin has been widely reported to have antimicrobial activity against a variety of pathogenic microorganisms, including viruses, bacteria, and fungi (Jenssen and Hancock, 2009; Superti, 2020; Gruden and Poklar Ulrih, 2021). The protein has a multifunctional mechanism of activity with both direct and indirect antimicrobial activities.

The direct antimicrobial activity of lactoferrin is generally thought to be dependent on two primary modes of action: iron chelation and microbial membrane disruption. Lactoferrin has two potent iron binding sites which allow for the molecule to chelate free iron and similar metal ions (Garcia-Montoya et al., 2012). This iron chelation exerts a broad-spectrum bacteriostatic activity which is generally unimpacted by antimicrobial resistance (Aguila et al., 2001). The protein is also highly glycosylated and positively charged, giving it highly varied macromolecular binding activities, including to key membrane components of gram-negative bacteria and certain yeast species (Drago-Serrano et al., 2012; Andres et al., 2016). In gram-negative bacteria, lactoferrin binds to lipopolysaccharide (LPS) which disrupts bacterial membranes to have a bactericidal efficacy as well as a host-protective anti-endotoxin effect (Appelmelk et al., 1994; Drago-Serrano et al., 2012). These activities not only limit bacterial growth but also potently reduce biofilm formation. This anti-biofilm efficacy is particularly strong as it draws on multiple aspects of the protein’s activity which can limit the metabolic activity specific to bacteria in the biofilm phenotype by sequestration of key nutrients, reduction of bacterial adhesion necessary for biofilm formation, and disruption of the extracellular matrix of bacterial biofilms (Singh et al., 2002; Ammons and Copie, 2013).

While the direct antimicrobial activity of lactoferrin has been extensively studied *in vitro*, the *in vivo* immunomodulatory activity of the protein also produces striking physiological effects independent of its direct antimicrobial efficacy in response to challenge (Haversen et al., 2003; Tanaka et al., 2021). Lactoferrin’s ability to sequester iron directly downregulates oxidative stress that commonly occurs during the immune response or physiological challenges (Liu et al., 2020). Lactoferrin also promotes the maturation of both macrophages and T-cells, preparing the immune system to respond to infection or other external challenges (Hu et al., 2017). Finally, lactoferrin suppresses the expression of pro-inflammatory cytokines and protects against toxin induced excessive inflammation by binding key pro-inflammatory factors produced during infections, such as LPS (Lutaty et al., 2020).

Lactoferrin has been tested for therapeutic potential for a broad range of diseases, including chronic wounds, cancer, oxidative stress, iron deficiency anemia, sepsis, infections, irritable bowel disease, pulmonary inflammation disorders, and hepatitis (Okada et al., 2002; Tsuda et al., 2002; Lyons et al., 2007; Tarnow-Mordi et al., 2020; El Amrousy et al., 2022; Zhao et al., 2022; Kaczynska et al., 2023). However, there have historically been major limitations to the commercial transition of lactoferrin. Most importantly, a naturally derived bovine product of sufficient purity and retained native bioactivity had not yet been produced or characterized due to a lack of industrial scale pharmaceutical grade production processes required to isolate and purify naturally derived full-length proteins without impacting the structure and functionality of the native molecule. Many protein engineering efforts using recombinant human or bovine lactoferrin sequences may not fully recapitulate native glycosylation essential to key aspects of lactoferrin bioactivity, such as its macromolecular binding activities. Lactea Therapeutics, and its affiliate Hyacinth Proteins, have developed a patent-pending process which can achieve ~100% purity and the complete retention of all identified functions of the molecule, referred to as Lactea Lf, including the protein’s broad-spectrum antimicrobial capabilities. This product is produced from raw chilled whole milk using APURA, a patent-pending high velocity diffusion technology designed for the chromatography of bioactive molecules from viscous feed materials with a high degree of specificity and sensitivity.

Based upon the antimicrobial activity of Lactea Lf and the interest of the United States Department of Defense to explore pathogen agnostic medical countermeasures, we tested Lactea Lf for its activity against several high consequence gram-negative bacterial pathogens and/or their respective surrogates. This strategy was used to determine if an agnostic medical countermeasure could be provided pre- and post-exposure to delay symptomatic onset until advanced detection and treatment could be provided. The Tier 1 gram-negative bacterial select agents we tested with Lactea Lf consisted of *Francisella tularensis* (causative agent of tularemia), *Burkholderia pseudomallei* (causative agent of melioidosis), and *Burkholderia mallei* (causative agent of glanders).

These bacterial pathogens pose a threat from both biodefense and public health perspectives as they have historically been developed as threat agents and are also endemic in the environment. In addition, approved vaccines are currently not available for any of these pathogens. The concern is further complicated with the possibility of the emergence of antibiotic resistant strains naturally, which would be much more likely with *B. pseudomallei* due to the presence of efflux pumps (Schweizer, 2012) or purposely being generated. The ability to derive resistance strains of *Francisella* or *Burkholderia* species is well documented in the literature with the potential to render current treatments ineffective (Dance et al., 1991; Loveless et al., 2010; Tandhavanant et al., 2010; Biot et al., 2013; Caspar et al., 2017; Chance et al., 2017; Heine et al., 2017; Biot et al., 2020; Superti, 2020).

In addition, *F. tularensis* and *B. pseudomallei* can form biofilms under *in vitro* conditions (Vorachit et al., 1995; Champion et al., 2019; Biot et al., 2020; Mlynek et al., 2022; Schaudinn et al., 2023; Bachert et al., 2021). Interesting, *B. mallei*, a strict host-adapted pathogen, has not been shown to readily produce this structure. For environmental bacteria, a biofilm would provide protection to the community to better survive adverse conditions, such as rapid osmolarity changes, nutrient deprivation, or even predation. Likewise, biofilm formation for pathogenic bacteria is typically considered a virulence factor, as it would allow the bacterial community the ability to withstand adverse events within a host, such as immune defense mechanisms, nutrient deprivation, and resistance to antibiotics. The exact role of biofilm in relation to virulence for both *F. tularensis* and *B. pseudomallei* is a current topic being debated (Taweechaisupapong et al., 2005; Mlynek et al., 2022). However, for *B. pseudomallei*, recent data suggests a correlation between biofilm formation in *B. pseudomallei* and virulence in mice by aerosol exposure (Cote et al., 2024). The goal of this study was to determine if Lactea Lf was able to ameliorate biofilm formation and/or growth inhibition of these bacterial pathogens or surrogate strains under *in vitro* conditions. Based upon the results, the study progressed to an *in vivo* murine model to determine if providing Lactea Lf could halt disease progression as measured by CFU recovery or prevent mice from succumbing to disease.

## Materials & methods

### Bacterial strains and culture conditions

A list of bacterial strains used in this study can be found in **Table 1**. *Burkholderia pseudomallei*, *Burkholderia thailandensis*, and *Burkholderia mallei* strains were cultured on blood agar plates (Remel) at 37°C or in liquid culture using brain heart infusion (BHI) broth (BSL-2 isolates) or LB broth supplemented with 4% glycerol (BSL-3 isolates). *Francisella tularensis* and *Francisella novicida* strains were cultured on chocolate agar plates (Remel) at 37°C or in liquid culture using BHI broth supplemented with 1% IsoVitaleX (Becton Dickinson) or Chamberlain’s defined medium (CDM) adjusted to pH 6.2 (Chamberlain, 1965).

**Table 1:** List of bacterial strains used in this study.

Strain	Strain description	Source
LVS	Live vaccine strain	USAMRIID collection
*F. novicida*	*Francisella novicida* (U112)	ATCC 15482
Schu S4	*Francisella tularensis* (type A)	BEI Resources (NR-10492) (Bachert et al., 2021)
OR96-0246	*Francisella tularensis* (type B)	BEI Resources (NR-648)
JW270	*Burkholderia pseudomallei* BSL-2 surrogate, DD503 derivative, Δ*wcb*; capsular polysaccharide mutant	USAMRIID collection (Warawa et al., 2009)
*B. thailandensis*	*Burkholderia thailandensis* (E264)	USAMRIID collection (Brett et al., 1998)
ATS2021	*Burkholderia pseudomallei*	CDC (Gee et al., 2022)
ATCC 23344	*Burkholderia mallei*	USAMRIID Collection (Nierman et al., 2004)

### Lactea Lf solution preparation

Lactea LF, an ultrapure form of bovine lactoferrin, (provided by Lactea Therapeutics, Frederick, MD, USA) was purified by its affiliate Hyacinth Proteins using APURA, a patent-pending high velocity diffusion technology designed to allow chromatographic purification of bioactive molecules from viscous feed materials with a high degree of specificity and sensitivity. The resulting Lactea LF is a ~100% pure form of bovine lactoferrin extracted from raw chilled whole milk and retains all identified functions of the molecule. Lactea Lf powder was resuspended in the medium indicated at a concentration of 100 mg/ml before use in static biofilm and growth inhibition assays.

### Static biofilm assay

Bacterial strains were cultured and resuspended to an OD_600_ equivalent to ~10^9^ CFU/ml in PBS. For *B. thailandensis* and *B. pseudomallei* JW270, cultures were further diluted to ~10^6^ CFU/ml in BHI. A Lactea Lf titration was made by 10-fold dilutions, from 100 mg/ml to 1 μg/ml, in BHI containing ~10^6^ CFU/ml of bacteria. Bacteria were seeded using 100 µl into a 96-well microtiter plate, in triplicate, to achieve an initial density of ~10^5^ CFU/well. For *B. pseudomallei* ATS2021 and *B. mallei* ATCC 23344, wells were seeded titratively using ~10^5–^10^7^ CFU/well in LB+4% glycerol. Finally, *F. novicida* and *F. tularensis* were seeded at ~10^6^ CFU/well in BHI or CDM.

Biofilm formation was measured after 24 hours for Burkholderia strains or 72 hours for *F. novicida* of static incubation at 37°C. Wells were aspirated and washed 3x with PBS to remove any non-adherent bacteria. The wells were fixed with 100% ethanol for 30 min. at room temperature. After ethanol fixation, 0.1% crystal violet (w/v) was added to each well for 15 min and subsequently washed 3x with PBS to remove excess crystal violet. Finally, 33% acetic acid was added to solubilize the remaining crystal violet and an OD_600_ reading was taken to quantify biofilm staining. When necessary, samples were diluted to ensure OD_600_ values were within the linear range of the instrument. Data shown are the average of three independent experiments.

### Growth inhibition assay

Strains were cultured on the appropriate agar plates for 24–48 hours and resuspended to an OD_600_ equivalent to 10^9^ CFU/ml in PBS. Strains were further diluted in the appropriate liquid culture medium. A Lactea Lf titration was made by 10-fold dilutions, from 100 mg/ml to 1 μg/ml, in media and seeded into a 96-well microtiter plate, in at least triplicate, with the indicated concentration of bacteria. Bacterial growth was measured by OD_600_ every 30 min. for 40 hours at 37°C shaking in a plate reader. Data shown are the average of at least 3 independent biological replicates.

### *In vivo* Lactea Lf treatment and murine challenges

Groups (n=16) of BALB/c mice (7–9 weeks old and obtained from Charles River Laboratories) were pretreated with Lactea Lf at varying doses (0, 25, 50, or 100 mg/ml in 25 µl of PBS) by intranasal delivery under mild injectable anesthesia (~0.15 ml per 20 g of body weight with a mixture of ketamine at 10 mg/ml, acepromazine at 1 mg/ml, xylazine at 2 mg/ml) daily beginning two days prior to challenge. On the day of challenge, mice were again treated with Lactea Lf and 30 min later challenged intranasally with a low dose of LVS (prepared from a chocolate agar plate) at the equivalent of <1 LD_50_. After challenge, Lactea Lf treatments were continued daily for 5 days and then stopped. An additional control group of mice received no intranasal treatment (PBS or Lactea Lf) and was only challenged with LVS.

To determine if treatment was able to provide a reduction in bacterial load from the lungs and spleens, these organs were collected from three mice from all groups at days 3 and 6 post-challenge. Mice were euthanized and immediately organs were sterilely collected, homogenized in PBS in a disposable tissue grinder (Covidien), serially diluted and plated on chocolate agar plates. Colonies were counted to determine CFU/organ. The remaining ten mice from each group were followed for 14 days post-challenge to determine if differences in survival would be observed. Mice were monitored several times each day and mortality rates (or euthanasia) were recorded. When required, euthanasia was performed in accordance with AVMA guidelines (Leary et al., 2020) using approximately 0.15 ml of Euthasol^®^ solution per 20 g of body weight.

Animal research at The United States Army of Medical Research Institute of Infectious Diseases (USAMRIID) was conducted and approved under an Institutional Animal Care and Use Committee (USAMRIID IACUC) in compliance with the Animal Welfare Act, Public Health Service Policy on Humane Care and Use of Laboratory Animals, and other federal statutes and regulations relating to animals and experiments involving animals. The facility where this research was conducted is accredited by the AAALAC, International and adheres to principles stated in *The Guide for the Care and Use of Laboratory Animals*, National Research Council, 2011.

### Lactea Lf protein tagging

Lactea Lf was labeled with either Oregon Green™ 488 Carboxylic Acid, Succinimidyl Ester, 5-isomer or Oregon Green™ 514 Carboxylic Acid, Succinimidyl Ester, (Thermo Fisher O6147, Thermo Fisher O6139). In brief, Lactea Lf was hydrated at 10mg/mL in a 0.1M sodium bicarbonate solution adjusted to pH 8.3, and the dyes were rehydrated at 10 mg/ml in DMSO. Next, 100 µL of the dye solution was slowly added to the protein solution while stirring. The solution was incubated at room temperature with continuous stirring. The protein was purified using Sephadex^®^ G, BioGel^®^ P in a 10 x 300mm spin column to separate the labeled protein for excess unlabeled dye. The protein’s bioactivity following labeling was validated using the QC process Hyacinth Proteins to validate protein bioactivity after purification.

### IVIS studies

Fluorescently tagged Lactea Lf (488 or 514) of a known concentration was serially diluted in sterile water and placed into a black 96-well plate for fluorescence measurement. The 96-well plate was imaged using the IVIS Spectrum CT (PerkinElmer) using the 540 nM emission filter and 500 nM excitation filter. Total radiant efficacy ([p/s]/[µW/cm²]) was quantified using the Living Image Software v4.5.4 (PerkinElmer) and drawing regions of interest (ROI) for each well on the plate.

Prior to Lactea Lf 488 administration, background tissue autofluorescence using the same emission and excitation filters used previously was measured by imaging anesthetized individual animals. Mice were administered Lactea Lf 488 under sedation intranasally in a volume of 10µL per nare to achieve the indicated concentrations. At 0.5h, 1h, 2h, 4h, and 6h post-administration, the mice were anesthetized with isoflurane (3-4%) delivered by an IMPAC^6^ machine (VetEquip) and imaged using an IVIS Spectrum CT instrument with the 540 nM emission filter and 500 nM excitation filter. Living Image Software v4.5.4 (PerkinElmer) was used to analyze the images. ROIs of identical sizes were used to calculate the total radiant efficiency for both the whole body and head only.

### Statistics

Analysis was implemented in SAS version 9.4 (SAS Institute Inc., Cary, NC). The IC_50_ was estimated with three-parameter or four-parameter Emax model. For each value a logistic growth curve was fit (Zwietering et al., 1990), which yielded estimates of max growth rate. AUC for each value was calculated with the method in paper “The SAS Calculations of Areas Under the Curve (AUC) for Multiple Metabolic Readings (Keh-Dong, 2002). Pairwise treatment groups were compared by negative binomial generalized linear model. No multiplicity adjustment was applied. The LVS challenged mice survival rates at selected time points were compared by Fisher exact test, and the times to death (TTD) were analyzed by Log-rank test for the pairwise comparison between the groups. A two-way ANOVA with Tukey’s multiple comparison applied *post-hoc* was used to compare fluorescent values in obtained from tissue.

## Results

### Lactea Lf inhibits *in vitro* biofilm formation of *Burkholderia* species in part by impacting growth

**Figure 1:**
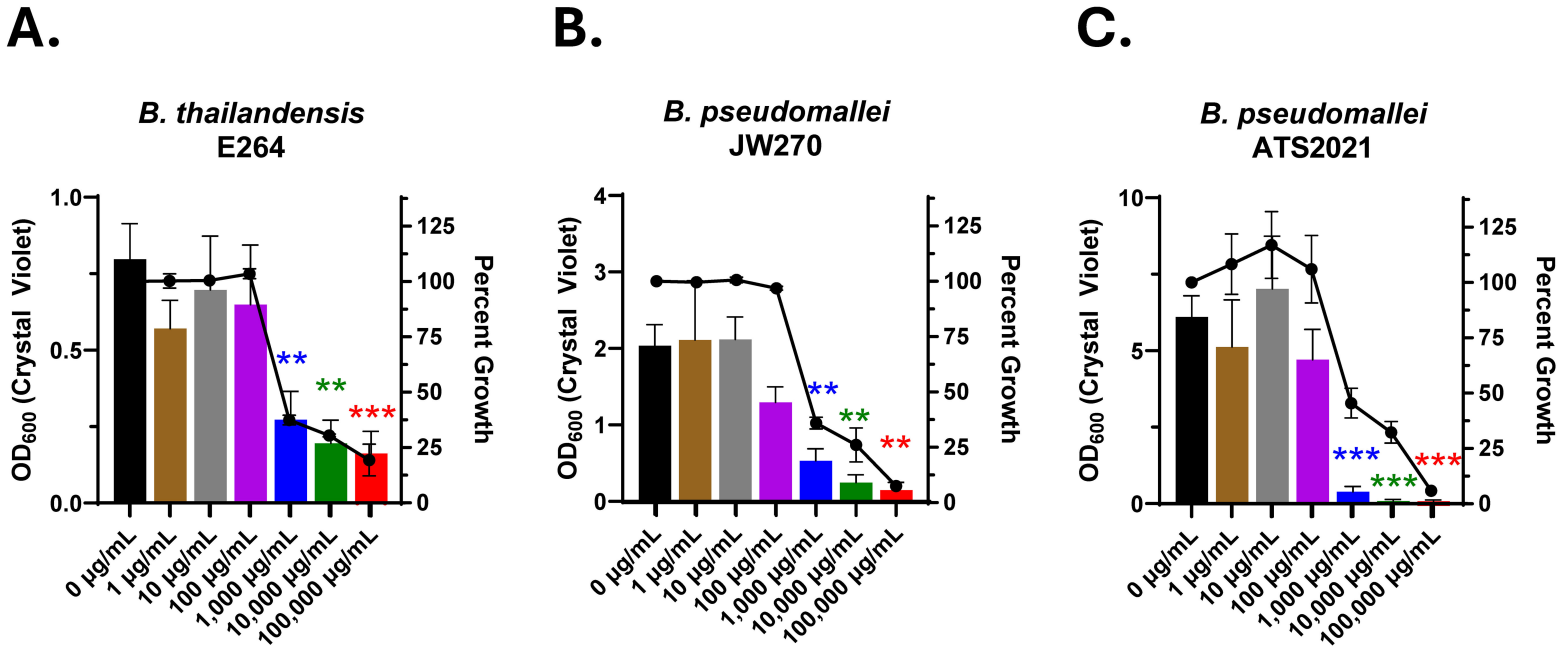
Biofilm formation of Burkholderia is inhibited in the presence of Lactea Lf. The BSL-2 surrogates **(A)** *B. thailandensis* and **(B)** *B. pseudomallei* JW270 or **(C)** virulent *B. pseudomallei* ATS2021 were seeded at 10^5^ CFU/well and cultured statically in the presence of Lactea Lf at the indicated concentrations. After 24h, planktonic bacteria were removed, and biofilm formation was quantified by crystal violet staining (left axis). Additionally, percent growth of each condition was calculated, using OD_600_, with respect to the growth of 0 μg/ml (right axis). Error bars represent the standard error of the mean from at least three independent experiments. **P<0.01; ***P<0.001.

The role of biofilm from a biodefense perspective is unclear, though it is well established that biofilm can affect treatment efficacy. From a preparedness standpoint, *Burkholderia* species represent the most likely biothreat where biofilm could present a challenge for patient treatment. We first assessed the effects of Lactea Lf on biofilm formation using three *Burkholderia* strains that exhibit different levels of biofilm formation *in vitro*. A soil-associated saprophyte, *B. thailandensis* generally forms a light pellicle biofilm at the air-liquid interface (Okaro et al., 2021). Testing Lactea Lf against *B. thailandensis* revealed that biofilm was inhibited beginning at approximately 1000 μg/mL (IC_50_ = 896.0 ± 130.0 μg/mL) and with little additional inhibition gained as the concentration increased (**Figure 1A**). *B. pseudomallei* JW270, a 1026b attenuated derivative that features a deletion of the *wcb* locus encoding 6-deoxyheptan capsular polysaccharide (Warawa et al., 2009), was tested next as this strain has been shown to make a moderate pellicle biofilm that is dependent upon eDNA (Okaro et al., 2022). *B. pseudomallei* JW270 displayed an increase in sensitivity to Lactea Lf (IC_50_ = 141.9 ± 129.7 μg/mL) that exhibited >90% inhibition at increased concentrations (**Figure 1B**). The third isolate tested was the fully virulent *B. pseudomallei* ATS2021 which has been shown to form a very robust pellicle biofilm (Cote et al., 2024). The calculated IC_50_ value for biofilm inhibition using Lactea Lf against this isolate was 187.2 ± 155.9 μg/mL (**Figure 1C**). We performed additional assays to determine if bacterial seed concentration (10^5–^10^7^ CFU) would alter the efficacy of Lactea Lf against *B. pseudomallei* biofilm as the amount of product to bacteria could alter the level of biofilm inhibition observed. No statistical differences were observed (**Supplementary Figure S1**). Additionally, the ability of Lactea Lf to disrupt biofilm after formation was assayed for *B. pseudomallei* ATS2021; however, no differences were observed suggesting that Lactea Lf was unable to affect this biofilm once established (data not shown).

**Figure 2:**
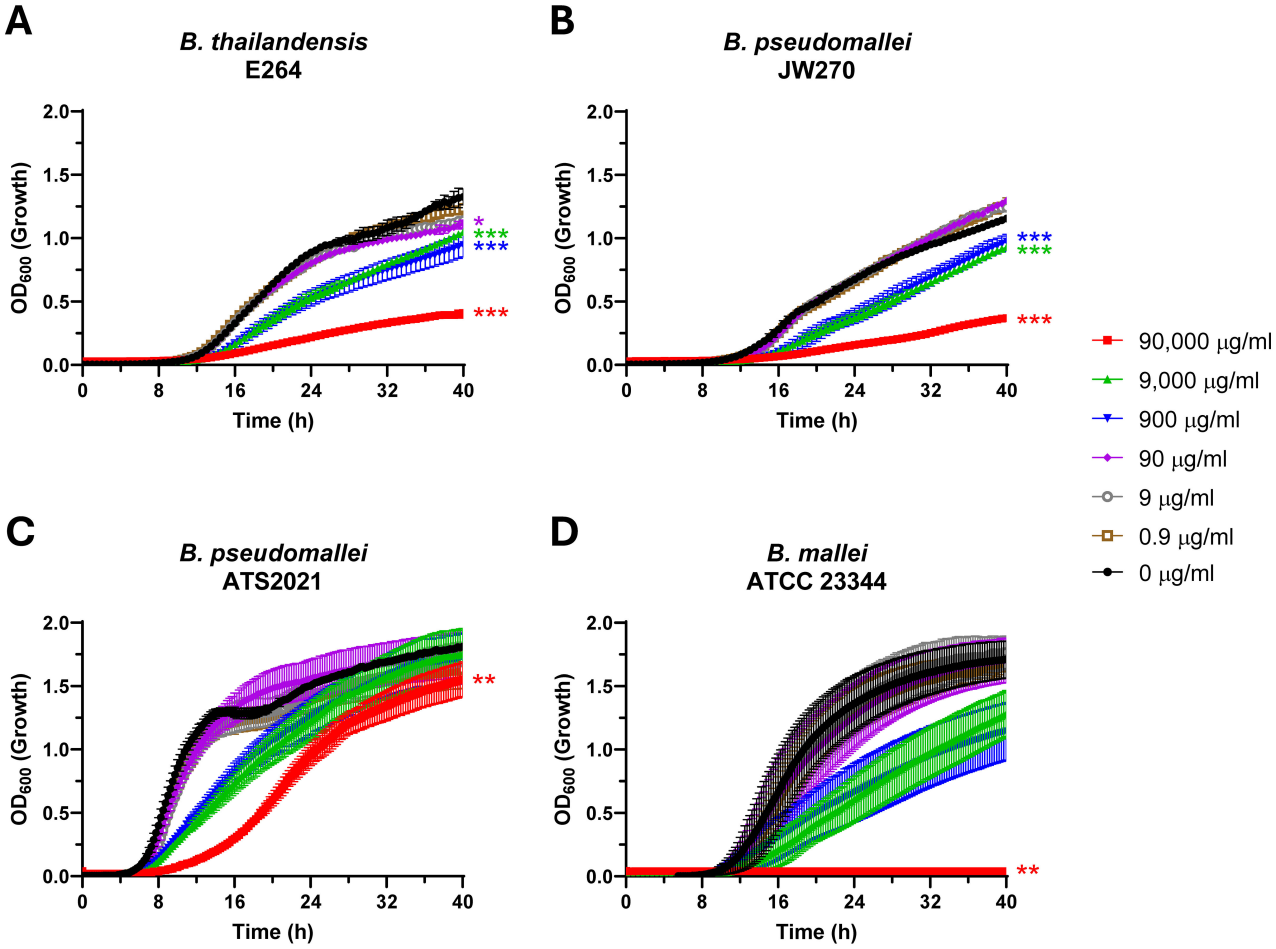
Lactea Lf impacts planktonic *in vitro* growth of Burkholderia in a dose-dependent manner. Dose response curves to Lactea Lf were generated for BSL-2 surrogates **(A)** *B. thailandensis* and **(B)** *B. pseudomallei* JW270 (seeded at 10^5^ CFU/well) as well as **(C)** virulent *B. pseudomallei* ATS2021 and **(D)** *B. mallei* ATCC 23344 (seeded at 10^6^ CFU/well) grown with shaking over the course of 40 h Growth was measured by OD_600_ for at least three technical replicates in each experiment. Error bars represent the standard error of the mean from three independent experiments. *P<0.05; **P<0.01; ***P<0.001.

Notably, growth was impacted by approximately 50% in the static biofilm assays. This prompted us to test the effects of Lactea Lf on *Burkholderia* species growth, and for these studies we also included *B. mallei* which does not readily form a biofilm. To accomplish this, growth curve analysis was performed with agitation to disrupt pellicle biofilm formation for *B. thailandensis* and *B. pseudomallei*. These experiments revealed that growth inhibition of *Burkholderia* by Lactea Lf generally occurs in a dose-dependent manner as expected but differs depending upon the bacterial species tested. The BSL-2 avirulent surrogates *B. thailandensis* and *B. pseudomallei* JW270 displayed inhibition beginning at 90 μg/ml and 900 μg/ml, respectively, while *B. pseudomallei* ATS2021 and *B. mallei* ATCC23344 were only significantly impacted at 90,000 μg/ml when analyzing the area of the curve (**Figure 2**; **Supplementary Table S1**). Further, growth inhibition was dependent upon initial inoculum density using *B. pseudomallei* ATS2021 under non-biofilm forming conditions (**Supplementary Figure S2**).

### Lactea Lf inhibits *in vitro* growth of *F. tularensis*

**Figure 3:**
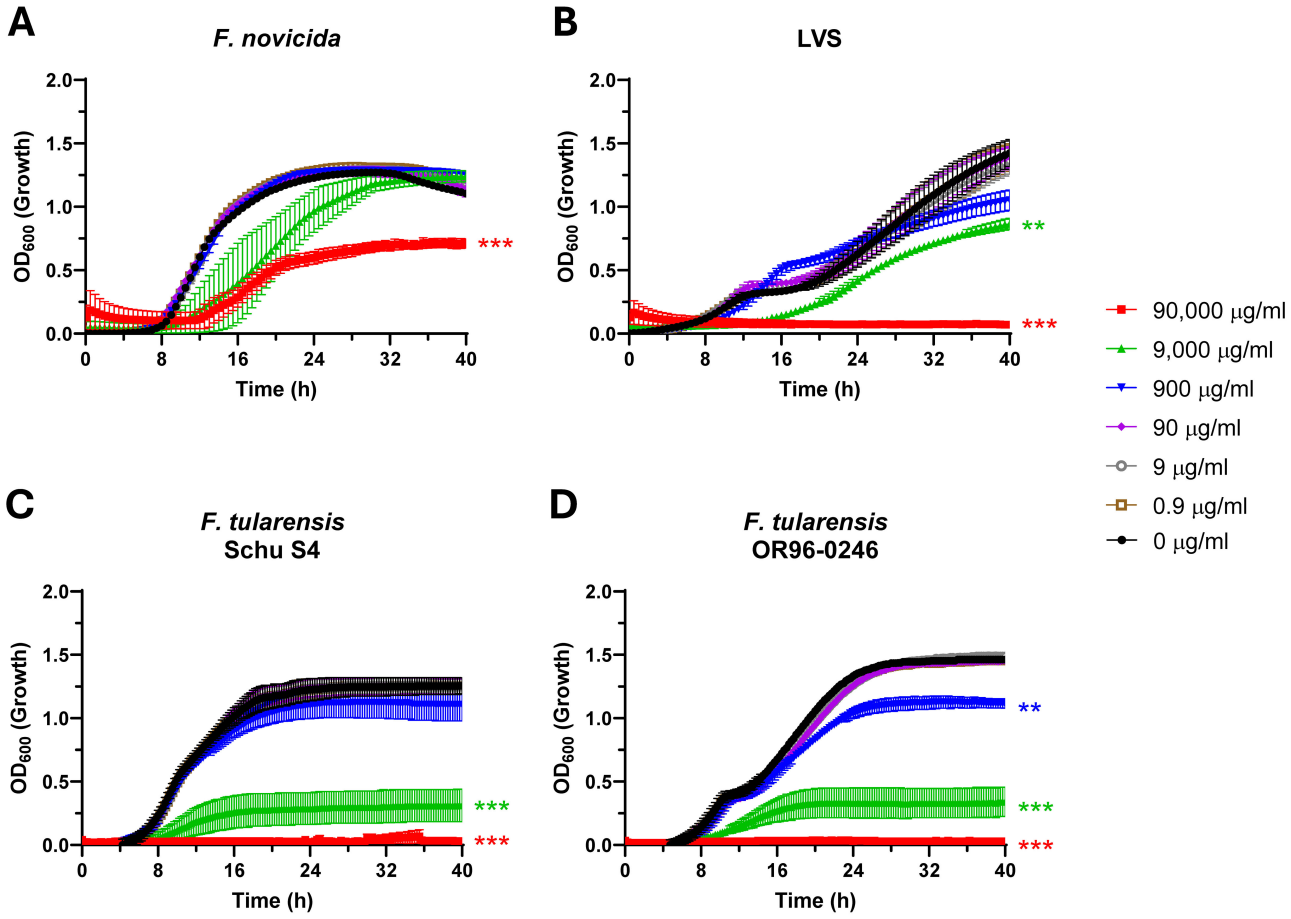
*F. tularensis* growth is inhibited in a dose-dependent manner in the presence of Lactea Lf in nutrient replete medium. Dose response curves to Lactea Lf were generated for **(A)** BSL-2 surrogates *F. novicida* and **(B)** *F. tularensis* LVS as well as **(C)** virulent *F. tularensis* Schu S4, and **(D)** *F. tularensis* OR-96243 (seeded at 10^7^ CFU/well) grown with shaking over the course of 40 h in CDM. Growth was measured by OD_600_ for at least three technical replicates in each experiment. Error bars represent the standard error of the mean from three independent experiments. **P<0.01; ***P<0.001.

Following the finding that Lactea Lf inhibited *Burkholderia* growth, work was expanded to include *Francisella* as this is a more fastidious bacterium. To accomplish this, we tested Lactea Lf against BSL-2 surrogates *F. novicida* and LVS as well as fully virulent *F. tularensis* SchuS4 (type A) and OR96-0246 (type B) in nutrient -replete CDM. Testing the surrogate strains, we found that Lactea Lf was able to only significantly alter the growth rate of *F. novicida* at 90,000 μg/ml (P<0.05, **Figure 3A**). LVS displayed increased sensitivity as significant inhibition was observed at 9,000 μg/ml of Lactea Lf (P<0.05, **Figure 3B**). However, complete inhibition was observed at this concentration for LVS as compared to only partial inhibition for *F. novicida*. Next, we tested Lactea Lf against two fully virulent *F. tularensis* strains and found the inhibition of the growth profile was comparable to *F. novicida*, with the exception that growth could also be 99% inhibited at the 90,000 μg/mL Lactea Lf concentration with a significant reduction in overall growth at 9,000 μg/ml (**Figures 3C, D**, **Supplementary Table S2**). These results suggest that Lactea Lf can potentially be effective against both type A and type B strains of *F. tularensis*.

**Figure 4:**
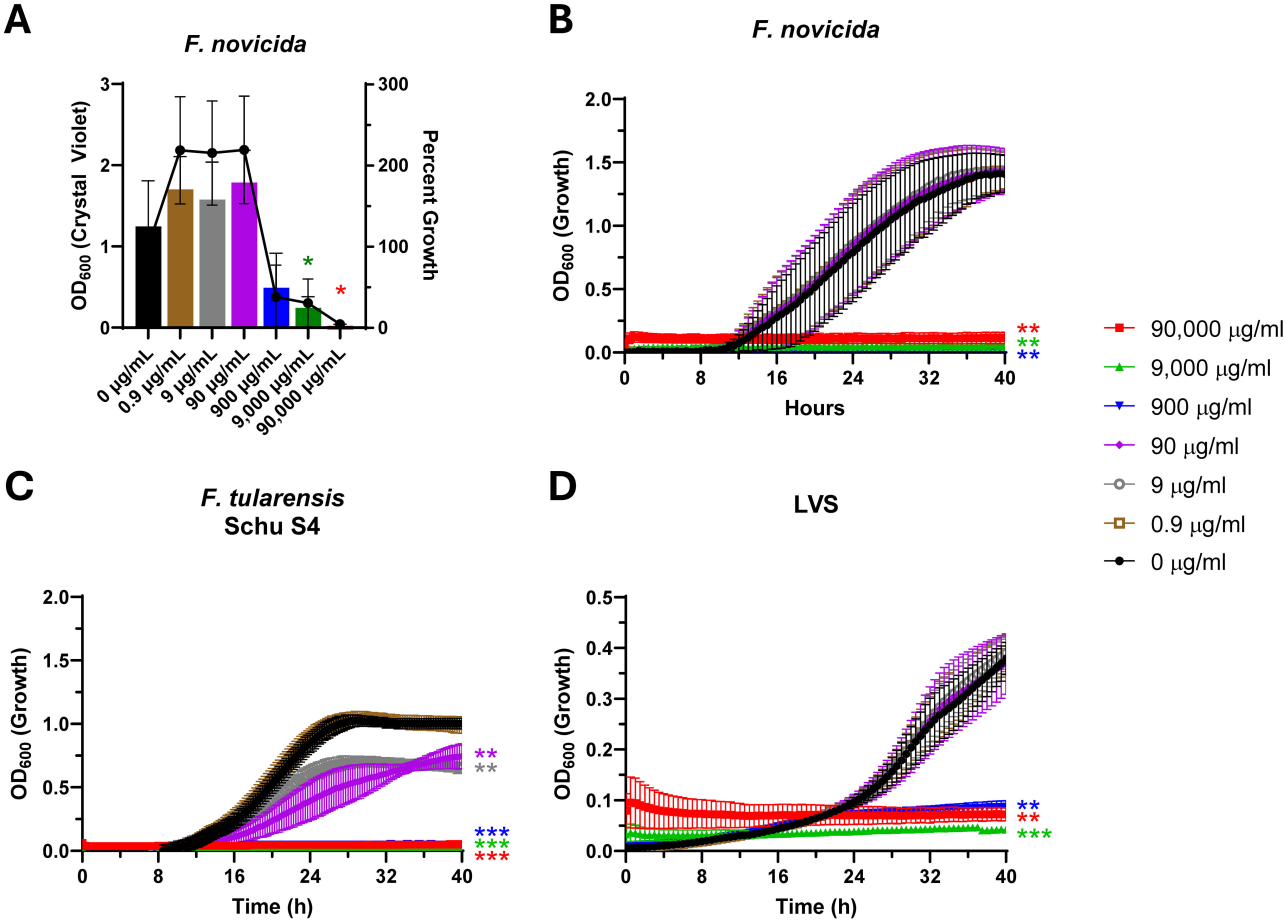
*F. novicida* biofilm formation and *F. tularensis* growth is inhibited in a dose-dependent manner with increased sensitivity observed in medium that mimics host-adaptation. The BSL-2 surrogate *F. novicida* **(A)** was seeded at 10^7^ CFU/well and cultured statically in the presence of Lactea Lf at the indicated concentrations. After 24h, planktonic bacteria were removed, and biofilm formation was quantified by crystal violet staining. Additionally, percent growth of each condition was calculated, using OD_600_, with respect to the growth of 0 μg/ml. Error bars represent the standard error of the mean from at least three independent experiments. Dose response curves to Lactea Lf were generated for **(B)** BSL-2 surrogate *F. novicida* and **(B)** as well as **(C)** virulent *F. tularensis* Schu S4, and **(D)** *F. tularensis* LVS (seeded at 10^7^ CFU/well) grown with shaking over the course of 40 h in BHI supplemented with 1% IsoVitaleX which is known to promote a host adapted state. Growth was measured by OD_600_ for at least three technical replicates in each experiment. Error bars represent the standard error of the mean from three independent experiments. *P<0.05; **P<0.01; ***P<0.001.

The growth medium is known to greatly impact the cellular state of *Francisella* as BHI has been shown to promote a host adapted phenotype (Hazlett et al., 2008) and permit biofilm formation in *F. novicida* (Margolis et al., 2010; Champion et al., 2019). While the role of biofilm formation in *F. tularensis* pathogenesis is unclear, we used *F. novicida* as a model to assess the potential of Lactea Lf for broad-spectrum anti-biofilm activity. This revealed biofilm inhibition of *F. novicida* was first observed at 900 μg/mL of Lactea Lf with an IC_50_ of 639 μg/mL and, unlike *B. pseudomallei*, appeared dependent upon the level of growth inhibition exhibited (**Figure 4A**).

In addition to being able to serve as an inhibitor of biofilm, the effectiveness of Lactea Lf in preventing growth of *F. tularensis* grown in BHI appeared to increase as significant inhibition was observed at 900 μg/mL for each isolate, with the fully virulent Schu S4 displaying sensitivity at 9 μg/mL (**Figures 4B–D**; **Supplementary Table S2**). Based on these data, we hypothesized this increase in sensitivity could provide a treatment window for *in vivo* efficacy model using the LVS strain of *F. tularensis* that is able to be performed under BSL-2 conditions.

### Lactea Lf in the current formulation was unable to protect in a murine tularemia challenge study using intranasal delivery

**Figure 5:**
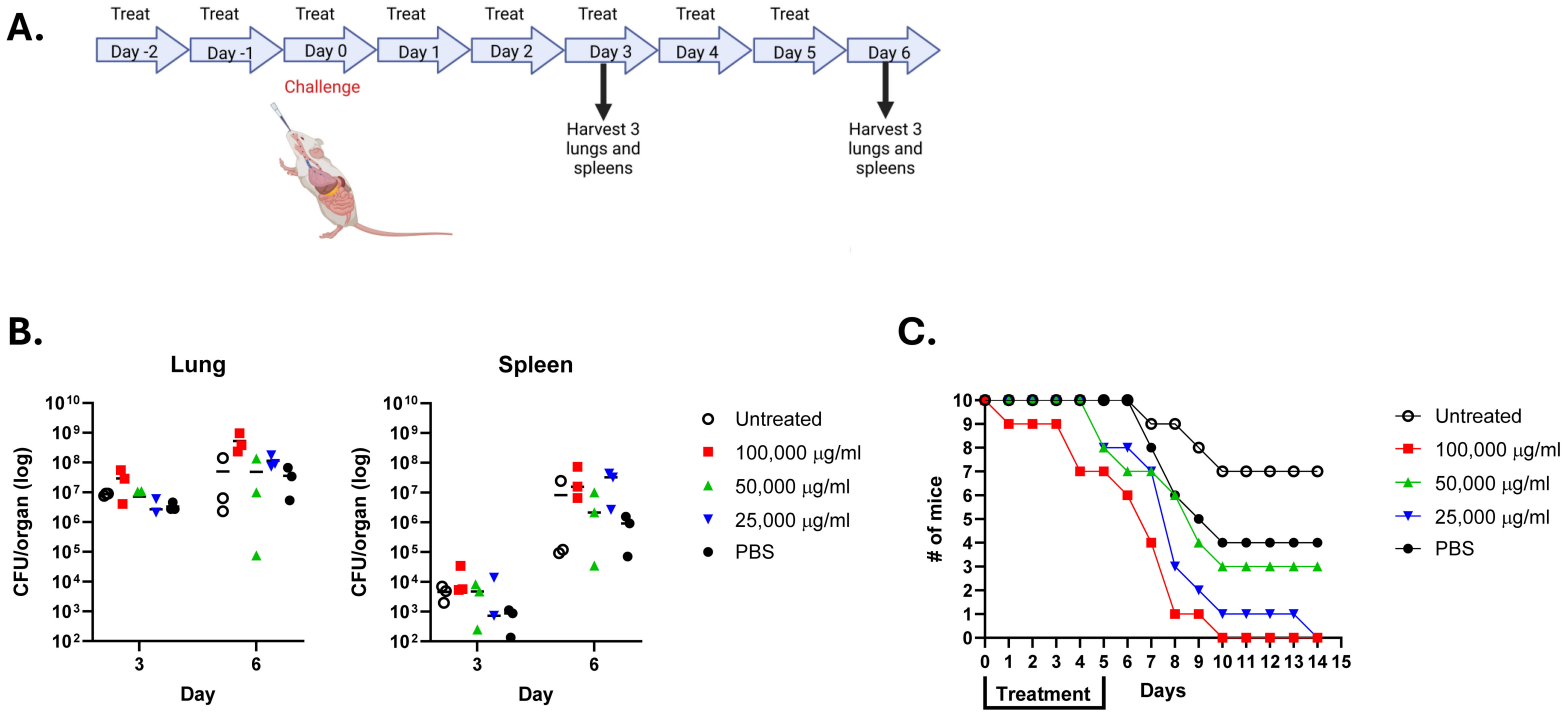
Lactea Lf does not protect mice challenged intranasally with *F. tularensis* LVS. Groups of mice (n=16) were provided the indicated amount of Lactea Lf intranasally 2 days prior to challenge through 5 days post challenge. Mice were challenged with approximately 1 LD_50_. **(A)** Schematic of the study design. Created with BioRender; Biryukov, S. (2025) https://BioRender.com/ras7ige. **(B)** At day 3 and 6 post challenge, the CFU present in the spleen and lung tissue from three mice determined for each dose. Bars represent the mean value for each treatment. **(C)** and the remaining 10 mice from each group were monitored for 21 days to record survivorship.

Based upon the *in vitro* results, we progressed the studies to determine if Lactea Lf treatment would provide some level of *in vivo* protection to BALB/c mice when exposed to a low pneumonic challenge dose of the *F. tularensis* LVS strain. With the goal of using Lactea Lf as a potential prophylactic as well as a disease mitigation strategy, mice were daily provided intranasally varying doses of Lactea Lf (25–100 mg/ml) for two days prior to challenge and then five days post-challenge (**Figure 5A**). This study contained two negative control groups of mice. For one of the treatment groups, mice received no Lactea Lf, just PBS alone. Another set of mice did not undergo the daily intranasal Lactea Lf treatments but was challenged with LVS to determine if the multiple intranasal instillations effected the outcome of the study.

To assess if Lactea Lf was able to inhibit dissemination of the *F. tularensis* and/or reduce the organ burden, bacterial CFUs were determined from the lungs and spleens from three mice taken on days 3 and 6 post-challenge. As shown in **Figure 5B**, no differences were found in the CFU counts recovered from the lungs for both days regardless of treatment. Likewise, similar results demonstrated an inability to prevent recovery of LVS CFUs from the spleens of Lactea Lf treated mice. In addition to determining CFU recovery from the organs, mice were followed for 14 days post-challenge to determine if Lactea Lf was able to protect the treated mice at this low challenge dose (**Figure 5C**). However, we were unable to see any level of protection to those mice provided Lactea Lf as compared to the untreated mice or those receiving only PBS.

### Biodistribution mapping of Lactea Lf through *in vivo* imaging

To understand whether the lack of *in vivo* protection observed for the *F. tularensis* LVS challenge was a product of the tissue distribution of Lactea Lf after intranasal installation, an *in vivo* imaging study was designed with fluorescently tagged Lactea Lf. Two different fluorescently tagged constructs, Lactea Lf 488 and Lactea Lf 514, were produced and evaluated *in vitro* to determine which fluorescent tag wavelength provides the brightest signal for *in vivo* imaging. The Lactea Lf 488 was slightly brighter than the Lactea Lf 514 (**Supplementary Figure S3**) and therefore was used for the *in vivo* biodistribution study.

**Figure 6:**
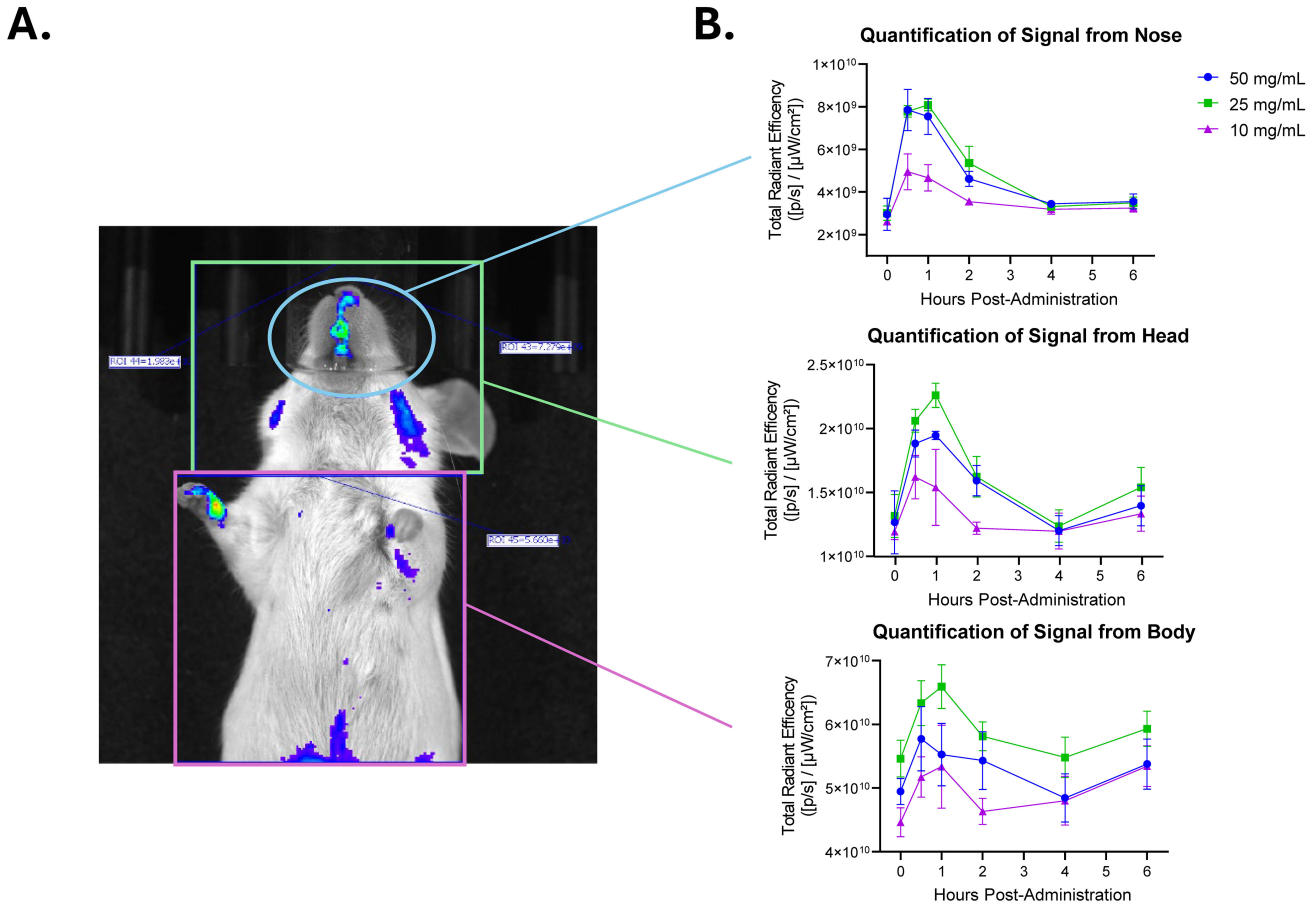
Fluorescently tagged Lactea Lf reaches peak intensity at 1 h post-administration when delivered intranasally. Naïve mice were administered fluorescent tagged Lactea Lf intranasally at the indicated doses and monitored using IVIS for 6 h. **(A)** A representative mouse administered with 50 mg/mL of Lactea Lf is shown at 1 h post administration. **(B)** Quantification of signal over time post administration. Data represent the average of three mice. Error bars represent the standard error of the mean.

**Figure 7:**
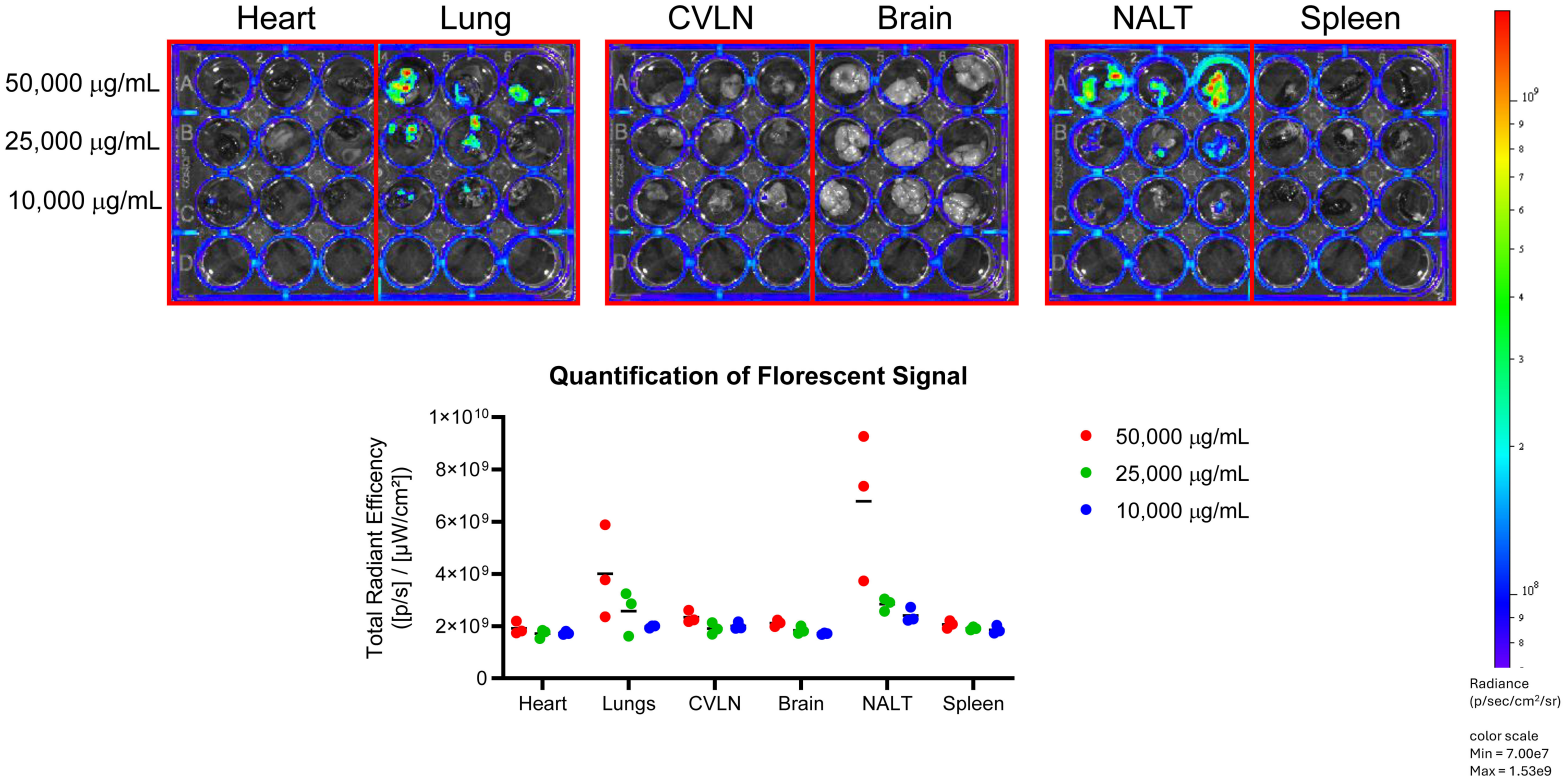
Lactea Lf localizes in the NALT post-administration of treatment. Tissues were harvested from mice administered fluorescent tagged Lactea Lf intranasally at 6 hours post-treatment and the fluorescent signal was viewed and quantified using an IVIS system. Data points represent the readings from the listed organs from three mice. Bar represents the average from the three data points.

Prior to the administration of Lactea Lf 488, all animals were imaged to collect background tissue autofluorescence data. Lactea Lf 488 was then administered intranasally at a concentration of 50, 25, or 10 mg/mL to mice (n=3/group), and images were collected using the IVIS Spectrum CT. Lactea Lf fluorescent signal was quantified in the animals over time from the nose, head and body (**Figure 6**). Signal in the animals peaked between 30 m and 1 h post administration of the Lactea Lf 488 and was more intense in the animals receiving 50 mg/mL and 25 mg/mL. Due to the rapidly declining signal just 6 hours after intranasal administration of Lactea Lf 488, tissues were harvested to quantify fluorescent signal *ex vivo*. Lactea Lf 488 fluorescent signal was still detectable in the lungs and nasal associated lymphoid tissue (NALT) in the animals treated with 50 mg/mL of Lactea Lf (P<0.05) and to a lesser extent the animals treated with 25 mg/mL (P>.05; **Figure 7**). These results suggest that Lactea Lf, in its present formulation and method of delivery, remained primarily confined to the intranasal region of the mouse including high binding to the NALT following intranasal delivery rather than being transported to the lungs which would be the major site of pneumonic infections.

## Discussion

One of the major innate immune defense mechanisms for mammals is lactoferrin. This protein is well established to have a broad spectrum of antimicrobial activity against a multitude of bacteria, fungi, and viruses, in addition to its potent immunomodulatory properties. For bacteria, lactoferrin has multiple antimicrobial properties that can affect growth and/or biofilm formation. Based upon the activity of lactoferrin, the goal of this study was to assess the potential of Lactea Lf, a novel, naturally derived and ultra purified version of bovine lactoferrin, as a medical countermeasure against multiple biothreat bacterial pathogens. The role of biofilm formation in human pathogenesis of bacterial biothreats is ill defined; however, it is important for preparedness against emerging threats to identify countermeasures that could eliminate biofilm as this bacterial lifestyle has been shown to be important for virulence in many other pathogenic bacterial species (Koo et al., 2017). The bacterial agents employed in this study were *F. tularensis*, *B. pseudomallei*, and *B. mallei* which all pose a threat to the public in endemic regions where these pathogens are present and to the military as these bacteria could be purposely used as a bioweapon (Christopher et al., 1997; Dance, 2000; Dennis et al., 2001; Khan and Ashford, 2001).

We initially determined that the anti-biofilm properties of Lactea Lf were capable of inhibiting its formation in *B. pseudomallei* (**Figure 1**) as this structure has been linked to virulence in animal studies when challenge occurs from aerosol exposure (Cote et al., 2024). These experiments showed that Lactea Lf could inhibit biofilm formation while also exerting some level of impact on growth. Our data are consistent with previous studies showing a chimeric peptide comprised of two antimicrobial domains of lactoferrin affected both growth and biofilm formation in *B. pseudomallei* (Puknun et al., 2013; Puknun et al., 2016). In addition, these results demonstrate that the purification of Lactea Lf from a natural source retains the expected effectiveness and functionality. However, mechanistically it remains unknown as to why Lactea Lf is able to elicit >99% biofilm inhibition in *B. pseudomallei*.

*Burkholderia* species have been shown to be affected by environmental iron levels (Caraher et al., 2007; Kamjumphol et al., 2013). Notably, iron chelation has mixed implications for biofilm depending upon the pathogen in question (Ammons and Copie, 2013; Rosa et al., 2017). For example, high environmental iron levels inhibit biofilm formation for *Legionella pneumophila* perhaps to promote dissemination in an effort to protect the cell against potential oxidative stress (Hindre et al., 2008). However, a case can be made that the more representative model is that iron is required for biofilm formation as it is an active process that requires cellular metabolism and, as such, iron availability typically is an important signal to regulate gene expression to control biofilm (Banin et al., 2005; Mey et al., 2005; Maresso and Schneewind, 2006; Wu and Outten, 2009). In *Pseudomonas aeruginosa* biofilm formation requires active iron transport, and it has been demonstrated that lactoferrin significantly restricts the development of biofilms in this species (Singh et al., 2002; Banin et al., 2005). These effects are attributed to apo-lactoferrin (iron free form of lactoferrin) since it has been shown to inhibit biofilm. In contrast, iron-saturated lactoferrin could cause aggregation that promotes biofilm (Berlutti et al., 2005). Berlutti et al. concluded that iron chelation by lactoferrin limited the biofilm form in *P. aeruginosa* and *B. cenocepacia*, particularly at sub-inhibitory concentrations as other iron chelators mirrored the observed effects.

The concentrations of Lactea Lf used in this study displayed ~50-70% growth inhibition in addition to biofilm inhibition which could impact other cellular processes in *Burkholderia*, such as quorum signaling. However, in our present study, we did not perform any measurements of quorum signaling. While little is known about the effects of quorum inhibition on *B. pseudomallei* biofilm formation, it has been established that two of the three N-acylhomoserine lactone (AHL) systems are required for full biofilm formation (Gamage et al., 2011). In a closely related pathogenic species of *Burkholderia*, it was demonstrated *B. cenopacia* cultured in high iron medium that supported biofilm formation also produced higher levels of AHL when compared to low iron medium in which biofilm formation was absent (Valenti et al., 2011). In this regard, it is possible that Lactea Lf partly controls biofilm formation by simply restricting the overall growth of Burkholderia, potentially limiting quorum signaling. However, this idea has not been tested in the present study.

The cationic charge of lactoferrin also begs to question if the biofilm matrix is directly altered as inhibition was assayed by the addition of Lactea Lf at the experimental onset. While much of this activity would be cell directed through LPS disruption, it has been noted that lactoferrin can disaggregate biofilm by degrading matrix components, particularly eDNA (Angulo-Zamudio et al., 2019). In our hands, we did not see that Lactea Lf was able to disrupt preformed biofilm by *B. pseudomallei* ATS2021, a strain that produces high amounts of biofilm. But it cannot be ruled out that matrix assembly was disrupted given our experimental design. *B. thailandensis* forms biofilms that are thought to be more dependent upon extracellular polysaccharides, but it is unclear what components comprise the *B. pseudomallei* ATS2021 biofilm (Okaro et al., 2021). We noted that *B. pseudomallei* JW270, which forms an eDNA based biofilm (Okaro et al., 2021), had no growth inhibition at 100 μg/mL of Lactea Lf, though biofilm was decreased by approximately 50% which would be consistent with direct effects on the biofilm matrix. While further experimentation is required, Lactea Lf may be more effective against pathogens that form an eDNA based biofilm which may be more efficiently inhibited.

Here, we have shown that Lactea Lf was able to alter the growth of *F. tularensis*, *B. pseudomallei*, and *B. mallei* adding to the list of pathogens that purified forms of lactoferrin can be employed against. The mechanism of action for lactoferrin could be bacteriostatic through metal chelation and/or bactericidal through peptide cleavage releasing lactoferricin (Tomita et al., 1991; Bellamy et al., 1992). Given that our growth data were generated by assaying the increase of turbidity overtime, it is likely that these data reflect iron depletion in culture rather than bactericidal activity. A consideration is that *Burkholderia* produce numerous siderophores and proteases that may be altered depending upon the various environmental factors. However, Lactea Lf was still able to inhibit the growth rate of *B. pseudomallei* at 900-9,000 μg/mL concentrations and completely inhibit *B. mallei* at the 90,000 μg/mL test concentration.

A future medical countermeasure directed against biofilm for *B. pseudomallei* would be best employed in conjunction with other treatments as the bacterium is highly resistant to several antibiotics and becomes more so when grown under conditions to induce biofilm formation (Sawasdidoln et al., 2010). And currently, despite treatment, melioidosis can become a chronic infection and has a propensity to remain latent with common relapses (White, 2003). A previous study has demonstrated a correlation between the *in vitro* ability of *B. pseudomallei* to form a biofilm and the relapse with human melioidosis cases (Limmathurotsakul et al., 2014). In addition, a previous study reports the presence of *B. pseudomallei* located within a biofilm in challenged animals and a human melioidosis patient (Vorachit et al., 1995).

The effectiveness of Lactea Lf against a third gram-negative biothreat agent, *F. tularensis*, was also assessed. In this study, we chose not to pursue the relationship between Lactea Lf and *F. tularensis* biofilm because pathogenic isolates do not readily form a biofilm, and when biofilm formation occurs the cell is an attenuated state (Mlynek et al., 2022). However, we did test *F. novicida* as this surrogate can form a robust biofilm (Durham-Colleran et al., 2010; Margolis et al., 2010; Zogaj et al., 2012) and found that Lactea Lf affected biofilm formation that appears to be dependent upon growth inhibition. We determined that the product was able to inhibit growth of both surrogate and fully virulent strains of *F. tularensis*, with the greatest affect seen when the bacteria were grown in supplemented BHI medium, which more closely resembles a host-like environment (Hazlett et al., 2008; Holland et al., 2017).

Based upon promising *in vitro* inhibitory effects with Lactea Lf, these studies progressed to a mouse challenge/treatment study. The use of various forms of lactoferrin have shown some promise as a therapeutic. For instance, a previous study demonstrated that treatment with a chimera form of lactoferrin and mice challenged with *Escherichia coli* O157:H7 was able to ameliorate damage following disease onset and reduced dissemination of bacteria (Flores-Villasenor et al., 2012). For our study described here, a murine pneumonic challenge model was employed with the LVS surrogate strain of *F. tularensis* to allow studies to be performed at BSL-2 while offering a greater window to gauge success as LVS displays a higher LD_50_ as compared to fully virulent strains (Fortier et al., 1991). Mice were pre-treated by intranasal delivery with varying levels of Lactea Lf and then challenged with a low dose of the LVS strain followed by five additional days of Lactea Lf treatment.

The concentrations of Lactea Lf used in this *in vivo* study would be in line with other published studies delivering lactoferrin to rodents (Bhimani et al., 1999; Yamauchi et al., 2000; Varadhachary et al., 2004; Takeuchi et al., 2006). However, as shown in **Figure 5**, we were unable to protect the challenged mice or ameliorate bacterial recovery from the lungs and spleens. Based upon the negative results observed with treatment with the LVS challenged mice, we did not expand the *in vivo* testing using Lactea Lf in its current form and challenges with fully virulent *F. tularensis* or more recalcitrant *B. pseudomallei* and *B. mallei*.

To determine possible reasons for the lack of any level of protection, the intranasal method of delivery was examined with fluorescently labeled Lactea LF. As shown in **Figure 6**, the primary localization of the Lactea Lf was in the NALT as opposed to the lungs which would be the predominant site for pneumonic tularemia (Heine et al., 2016). Furthermore, the half-life of the fluorescent signal at any of the sites where Lactea Lf was deposited peaked at about 30 min – 1 h following delivery and then was quickly cleared. These results also suggest that Lactea Lf may be trafficked from the site of deposition and/or be degraded as was previously observed by other routes of lactoferrin delivery (Ji et al., 2006; Takeuchi et al., 2006).

Despite these current limitations from this preliminary study, further consideration should be given for Lactea Lf as potential treatment for biothreat bacterial pathogens with additional improvements. As shown with the current formulation, intranasal delivery may not be the best route. Lactea Lf could potentially be better suited as a prophylactic countermeasure delivered via aerosol. Lactea Therapeutics is currently testing the tolerability and biodistribution of nebulized Lactea Lf using a mouse model to evaluate whether this may be a better delivery mechanism for these kinds of studies. Optimization of this delivery may be crucial for the further evaluation and translation of Lactea Lf in pulmonary applications. In addition, development of improved pulmonary delivery may additional enable Lactea Lf to translate in the treatment of chronic lung bacterial infections, including in cystic fibrosis patients which has showed great promise in prior literature (Cutone et al., 2019) using both *in vitro* and *in vivo* models. Furthermore, additional methods or formulations could be explored for the stabilization of Lactea Lf.

From this study, we only examined the use of Lactea Lf as a singular therapy against highly pathogenic bacteria. Potentially, Lactea Lf could be used as part of a combinational/concurrent therapy. In previous work, it was shown that the sensitivity to various antibiotics for *B. cepacia* and *P. aeruginosa* could be greatly increased in the presence of lactoferrin (Alkawash et al., 1999). Similar increases in antibacterial sensitivities were observed in co-dosing studies with lactoferrin for additional other bacterial pathogens (Ellison et al., 1990; Naidu and Arnold, 1994) demonstrating the agnostic potential of this therapy. A second potential consideration for future use of Lactea Lf would be to apply the “LIMIT” (Layered and Integrated Medical Intervention Technologies) approach by including it with other medical countermeasures. A “layered” approach is defined as multiple medical countermeasures that are delivered at distinct times. Fortuitously, this approach has allowed previous therapeutics and/or vaccines which have only been studied independently and may have performed suboptimally to be reexamined with promising results. The layered approach has allowed some of these products to work significantly better against various biothreat pathogens when evaluated with the addition of other countermeasures (Aardema et al., 2005; Vietri et al., 2006; Cote et al., 2021; Klimko et al., 2022a; Klimko et al., 2022b; Vagima et al., 2022; Biryukov et al., 2023; Barnes et al., 2024; Davies et al., 2024).

Our objective for this study was to determine if Lactea Lf was able to have any impact on the fitness of these biothreat bacterial pathogens, and if so, could we improve the overall disease outcomes in an animal model with providing this pathogen agnostic antimicrobial product. To our knowledge, no specific work has been previously completed with the interaction of lactoferrin and *F. tularensis* making these results of our study novel. As shown in **Figures 3** and **4**, *F. tularensis* growth and *F. novicida* biofilm formation was inhibited. However, several previous publications have studied various interactions of lactoferrin in its native and chimera (combination of its two antimicrobial domains) with several of the pathogenic *Burkholderia* species, including *B. pseudomallei* (Puknun et al., 2013; Puknun et al., 2016). Interestingly, to our knowledge *B. mallei* has not been included in any such studies. As shown in **Figure 2**, Lactea Lf was able to have a similar inhibitory effect on *B. mallei*.

Although Lactea Lf was unable to treat mice challenged with LVS, there is the possibility for the use of it as a medical countermeasure against biothreat agents. Intranasal delivery was chosen as the preferred method of delivery in this experiment due to the assumption it would be transported to the lung environment. Based on the predominant localization of the fluorescently labeled Lactea Lf in the NALT as opposed to the lungs and its short duration where it was deposited, we speculate that intranasal delivery may not be the best route in the current formulation. Lactoferrin is widely thought to have relatively high tolerability, which has been thoroughly characterized for oral delivery *in vivo* resulting in the protein being recognized as safe in certain oral applications at acceptable doses in both America and Europe from a regulatory perspective (Yamauchi et al., 2000). As such, we propose that Lactea Lf could potentially be better suited as a prophylactic countermeasure or used as a part of a combination therapy, although future studies may have to further examine the pulmonary tolerability of Lactea Lf in humans if the protein will be delivered directly by aerosol in a final application. Successful translation of these efforts could have a substantial impact on both biodefense preparedness and be a great public health benefit if the protein can further translate to treat lung disease and infections (Kaczynska et al., 2023). Future studies will include looking at different formulations of Lactea Lf, different methods of delivery, and testing prophylactic and combination therapy treatment regiments. Once identified, future efforts could be focused on the immunomodulation properties of Lactea Lf to protect against additional pathogens beyond bacteria to include viruses to provide an agnostic medical countermeasure.

## Author’s note

The opinions, interpretations, conclusions, and recommendations presented are those of the authors and are not necessarily endorsed by the U.S. Army or Department of Defense. The use of either trade or manufacturers’ names in this report does not constitute an official endorsement of any commercial products. This report may not be cited for purposes of advertisement. This research was supported in part by an appointment to the Department of Defense (DoD) Research Participation Program administered by the Oak Ridge Institute for Science and Education (ORISE) through an interagency agreement between the U.S. Department of Energy (DOE) and the DoD. ORISE is managed by Oak Ridge Associated Universities (ORAUs) under DOE contract number DE-SC00014664. All opinions expressed in this document are the author’s and do not necessarily reflect the policies and views of DoD, DOE or ORAU/ORISE. Team Chenega (EM and CG), Laulima Government Solutions (JQ) - Contractors. This does not constitute an endorsement by the US Government of these or any other contractor.

## Data Availability

The original contributions presented in the study are included in the article/**Supplementary Material**. Further inquiries can be directed to the corresponding author.
