# Supplementary material for: Treatment of bacterial biothreat agents with a novel purified bioactive lactoferrin affects both growth and biofilm formation
Source: Front Cell Infect Microbiol. 2025 Jun 17;15:1603689. doi: 10.3389/fcimb.2025.1603689 (PMC12209266; doi:10.3389/fcimb.2025.1603689)
Supplement: Supplementary file 1 [file DataSheet1.docx]

**Treatment of Bacterial Biothreat Agents with a Novel Purified Bioactive Lactoferrin Affects both Growth and Biofilm Formation**

Christian J. Xander^1*^, Elsie E. Martinez^1*^ Ronald G. Toothman^1^, Christina L. Gardner^2^, Ju Qiu^3^, Jonathan Snedeker^4^, Matthew H. Bender^4^, Christopher Hlubb^4^, Crystal W. Burke^2^, Joel A. Bozue^1^, and Kevin D. Mlynek^1π^

^1^Bacteriology Division, ^2^Virology Division, ^3^Regulated Research Administration Division U.S. Army Medical Research Institute of Infectious Diseases (USAMRIID), Frederick, MD, USA, ^4^Lactea Therapeutics, Frederick, MD, USA

Keywords: Francisella, Burkholderia, biofilm, lactoferrin, tularemia, melioidosis, glanders

^π^ corresponding author: kevin.d.mlynek.civ@health.mil

*Authors equally contributed to this study

**Supplemental Figures:**


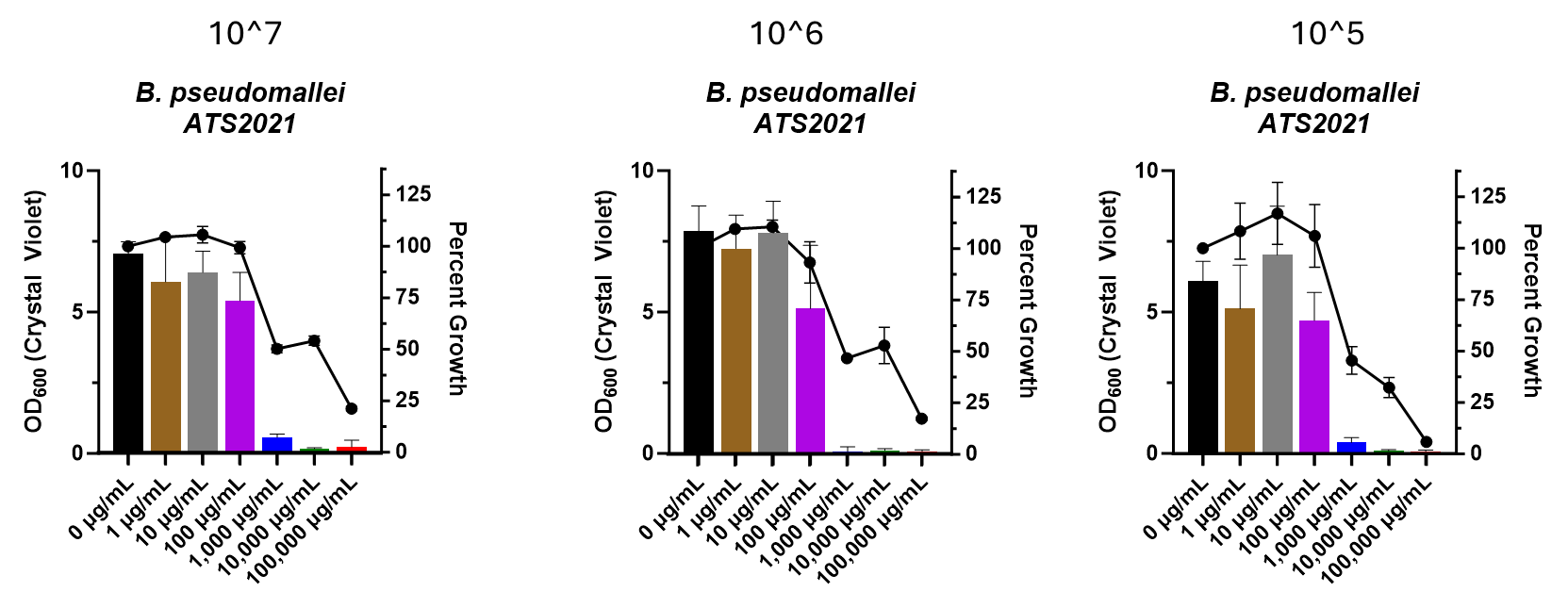


**Supplementary Figure 1. Biofilm inhibition is not dependent upon initial seeding density.** *B. pseudomallei* ATS2021 was cultured statically in the presence of Lactea Lf at the indicated concentrations using several starting concentrations of bacteria. After 24h, planktonic bacteria were removed, and biofilm formation was quantified by crystal violet staining (left axis). Additionally, percent growth of each condition was calculated, using OD_600_, with respect to the growth of 0 μg/ml (right axis). Error bars represent the standard error of the mean from at least 3 independent experiments.


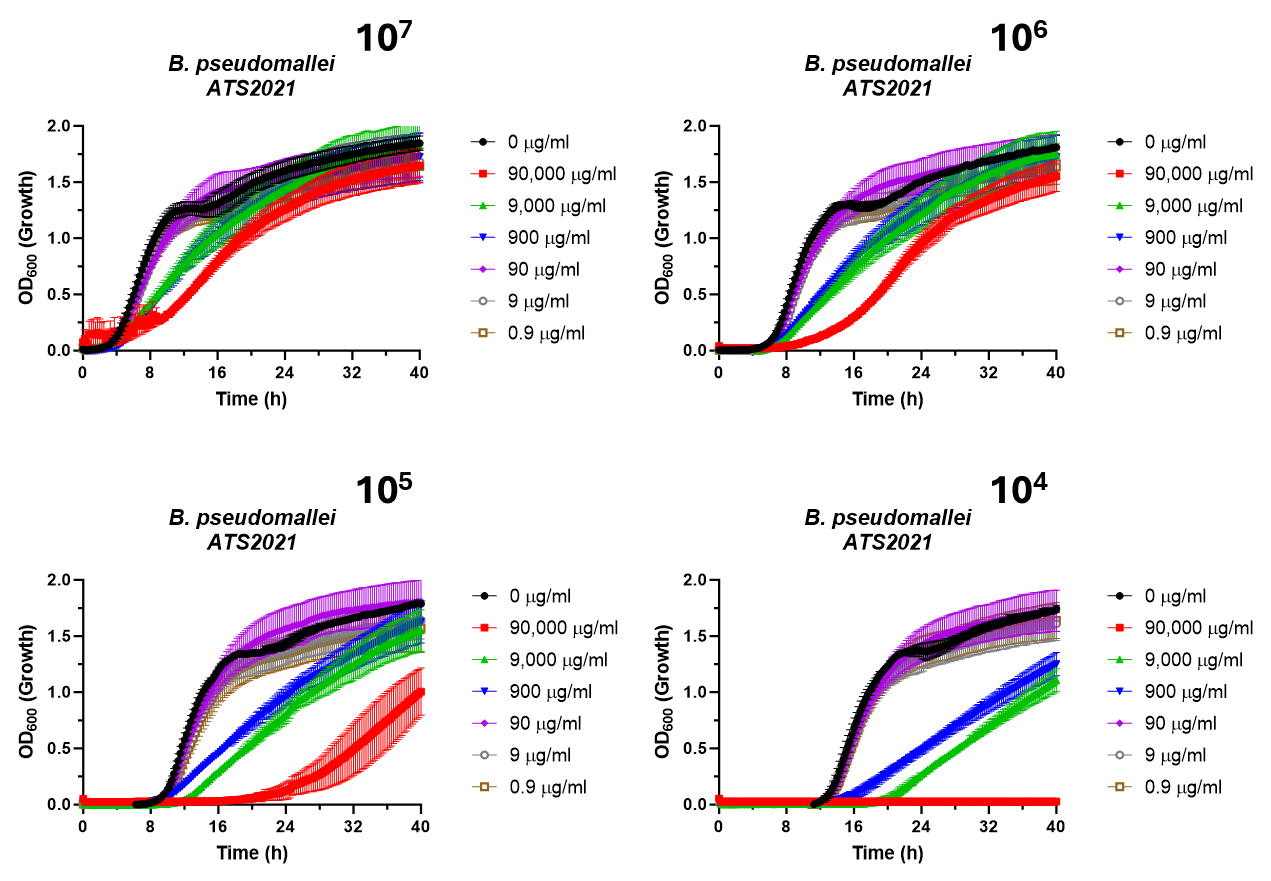


**Supplementary Figure 2. Growth inhibition of *Burkholderia* by Lactea Lf is affected by starting density.** *B. pseudomallei* ATS2021 was cultured with shaking in LBG in the presence of Lactea Lf at the indicated concentrations. Growth was monitored by OD_600_ measurements over the course of 40 h. Error bars represent the standard error of the mean from at least 3 independent experiments.


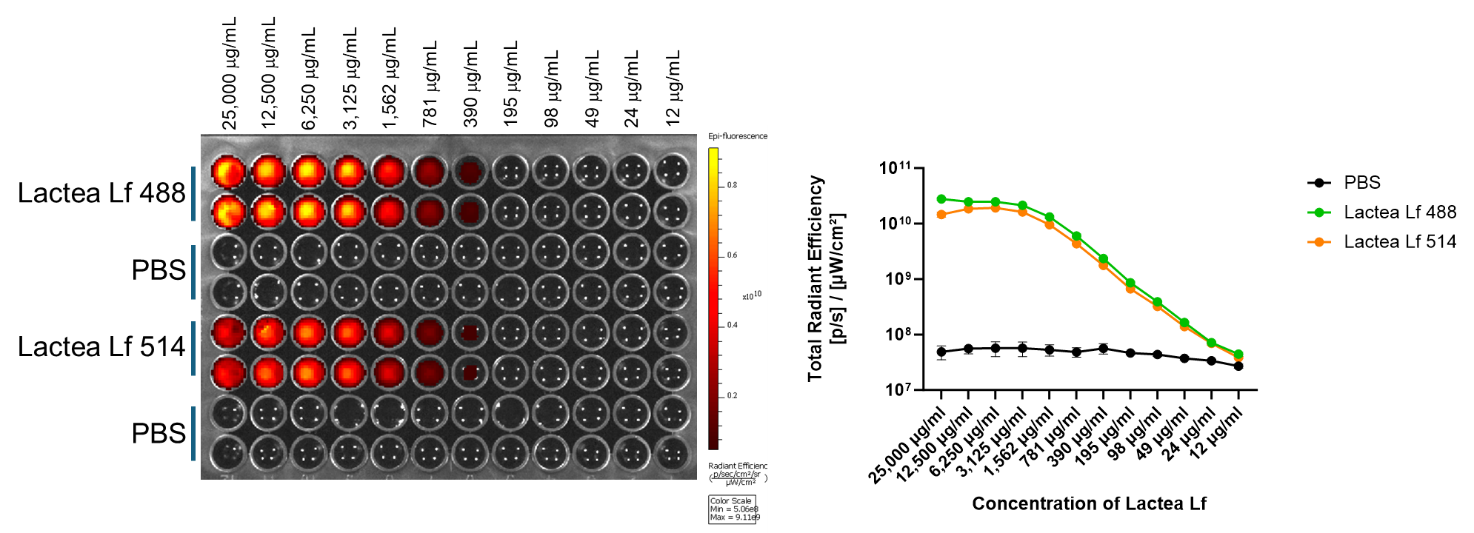


**Supplementary Figure 3. Lactea Lf 488 labeled product provides a slightly stronger signal detection by IVIS.** Serially dilutions of fluorescently labeled Lactea Lf was imaged using IVIS to determine the optimal protein to use in bioavailability assays performed in mice studies.

**Supplemental Tables:**

**Supplementary Table 1: Statistical analysis of *Burkholderia* growth in the presence of Lactea Lf.**

|  | | ***B. thailandensis* E264** | ***B. pseudomallei* JW270** | ***B. pseudomallei* ATS2021** | ***B. mallei* ATCC 23344** |
| --- | --- | --- | --- | --- | --- |
| **Max Growth Rate (OD600/h)** | vs. 90,000 μg/ml | **<0.0001** | **<0.0001** | **0.0417** |  |
|  | vs. 9,000 μg/ml | **0.0045** | 0.8720 | **0.0352** | 0.1094 |
|  | vs. 900 μg/ml | **0.0006** | 0.9449 | **0.0499** | 0.1309 |
|  | vs. 90 μg/ml | **0.0094** | 0.2303 | 0.8308 | 0.9577 |
|  | vs. 9 μg/ml | **0.0176** | 0.3717 | 0.5296 | 0.8312 |
|  | vs. 0.9 μg/ml | 0.2154 | 0.2954 | 0.5612 | 0.6655 |
| **AUC Total** | vs. 90,000 μg/ml | **<0.0001** | **<0.0001** | **0.0014** | **0.0025** |
|  | vs. 9,000 μg/ml | **0.0004** | **0.0004** | 0.0572 | 0.0897 |
|  | vs. 900 μg/ml | **0.0002** | **0.0007** | 0.0742 | 0.0974 |
|  | vs. 90 μg/ml | **0.0287** | 0.2454 | 0.8993 | 0.8099 |
|  | vs. 9 μg/ml | 0.0526 | 0.3848 | 0.4750 | 0.9904 |
|  | vs. 0.9 μg/ml | 0.3627 | 0.3727 | 0.3744 | 0.7059 |

|  | | ***F. novicida* U112 in CDM** | ***F. tularensis* LVS in CDM** | ***F. tularensis* Schu S4 in CDM** | ***F. tularensis* OR96-0246 in CDM** | ***F. tularensis* LVS in BHI** | ***F. tularensis* Schu S4 in BHI** |
| --- | --- | --- | --- | --- | --- | --- | --- |
| **Max Growth Rate (OD600/h)** | vs. 90,000 μg/ml | **<0.0001** | **0.0030** | **<.0001** | **<.0001** | 0.3744 | **<0.0001** |
|  | vs. 9,000 μg/ml | **0.0049** | 0.6532 | **<.0001** | **<.0001** | 0.3743 | **<0.0001** |
|  | vs. 900 μg/ml | 0.7510 | 0.4021 | 0.4430 | 0.1032 | 0.3744 | **<0.0001** |
|  | vs. 90 μg/ml | 0.5276 | 0.7951 | 0.8585 | 0.6778 | 0.6913 | **0.0515** |
|  | vs. 9 μg/ml | 0.7365 | 0.7823 | 0.8873 | 0.7074 |  | **0.0396** |
|  | vs. 0.9 μg/ml | 0.4968 | 0.8884 | 0.9742 | 0.7378 | 0.9966 | 0.8382 |
| **AUC Total** | vs. 90,000 μg/ml | **0.0009** | **<0.0001** | **<0.0001** | **<0.0001** | **0.0074** | **<0.0001** |
|  | vs. 9,000 μg/ml | 0.1592 | **0.0054** | **<0.0001** | **<0.0001** | **0.0004** | **<0.0001** |
|  | vs. 900 μg/ml | 0.4825 | 0.3474 | 0.2940 | **0.0020** | **0.0020** | **<0.0001** |
|  | vs. 90 μg/ml | 0.6669 | 0.8928 | 0.9729 | 0.4204 | 0.9763 | **0.0015** |
|  | vs. 9 μg/ml | 0.7744 | 0.9339 | 0.9433 | 0.5956 | 0.8379 | **0.0044** |
|  | vs. 0.9 μg/ml | 0.5266 | 0.9857 | 0.9455 | 0.6446 | 0.8554 | 0.9997 |

**Supplementary Table 2: Statistical analysis of *Francisella* growth in the presence of Lactea-Lf.**
